# Deep learning and taphonomy: high accuracy in the classification of cut marks made on fleshed and defleshed bones using convolutional neural networks

**DOI:** 10.1038/s41598-019-55439-6

**Published:** 2019-12-12

**Authors:** Gabriel Cifuentes-Alcobendas, Manuel Domínguez-Rodrigo

**Affiliations:** IDEA (Institute of Evolution in Africa), University of Alcalá de Henares, Covarrubias 36, 28010 Madrid, Spain

**Keywords:** Machine learning, Anthropology

## Abstract

Accurate identification of bone surface modifications (BSM) is crucial for the taphonomic understanding of archaeological and paleontological sites. Critical interpretations of when humans started eating meat and animal fat or when they started using stone tools, or when they occupied new continents or interacted with predatory guilds impinge on accurate identifications of BSM. Until now, interpretations of Plio-Pleistocene BSM have been contentious because of the high uncertainty in discriminating among taphonomic agents. Recently, the use of machine learning algorithms has yielded high accuracy in the identification of BSM. A branch of machine learning methods based on imaging, computer vision (CV), has opened the door to a more objective and accurate method of BSM identification. The present work has selected two extremely similar types of BSM (cut marks made on fleshed an defleshed bones) to test the immense potential of artificial intelligence methods. This CV approach not only produced the highest accuracy in the classification of these types of BSM until present (95% on complete images of BSM and 88.89% of images of only internal mark features), but it also has enabled a method for determining which inconspicuous microscopic features determine successful BSM discrimination. The potential of this method in other areas of taphonomy and paleobiology is enormous.

## Introduction

Deep learning (DL) is the most advanced branch of machine learning (ML), which is at the foundation of artificial intelligence (AI). Recently, ML algorithms have provided higher rates of accuracy in the classification of bone surface modifications (BSM) from controlled experiments than traditional statistical methods^1–3^. DL algorithms are powerful at many classification tasks, but they currently are the best method for computer vision, through image identification and classification. Neural networks make up the core of DL analyses. There is a diverse array of neural network topologies (i.e., recurrent, gated recurrent, feed forward, long/short term, auto encoder, variational, Markov Chain, Hopfield, Boltzmann, deep belief, liquid state machine, Kohonen, deep residual, neural Turing, deconvolutional, generative adversarial). Among these, deep convolutional neural networks (DCNN) currently are some of the most successful at image identification. A publication of the first use of computer vision in the field of taphonomy, showed how the use of DCNN on an experimental set of bone surface modifications, involving cut marks made with simple and retouched stone flakes and trampling marks, produced accuracy rates of correct classification >91% (>50% higher than human expert assessments)^1^.

This new objective approach to the study of BSM can allow discernment of important controversial issues surrounding BSM. Taphonomists have long debated whether experimental BSM generated on fresh bones are adequate proxies for prehistoric BSM. For instance, Gaudzinski-Windheuser *et al*.^4^ argued that prehistoric cut marks result from longitudinal transformation of their microscopic features caused by palimpsestic superimposition of processes. This is rarely considered in experimentation. BSM are subjected to the morphing of their microscopic features due to this dynamic process^5,6^. Cut mark variability can also be impacted by rock type and bone portion^7^. It has also been argued that experimental butchery conditions determine cut mark diversity and properties; among these, it is argued that cut marks imparted on defleshed bone have different properties from (and therefore, cannot be mistaken with) cut marks resulting from bulk defleshing^8^. The heuristics of these statements depend on the analytical approach. BSM identification remains a subjective endeavour, given that the categorization of variables change according to the analyst^9^, and that interpretations on BSM variability are usually based on geometric and metric approaches that are so extremely variable that the same actor-effector-trace can produce statistically significant differences within the same sample^10^. Cut marks may vary metrically depending on bone portion and tool if using simple bivariate (depth-width) methods^8^ or not, if using multivariate geometric-morphometric methods^11^. Regardless, if metric variability does not impact on microscopic properties of BSM, these can be accurately identifiable if the analytical method is based on such properties^10^.

Irrespective of the approach, one of the main questions is whether cut marks experimentally generated on defleshed bones and those made through bulk defleshing can be differentiated (given their variable diversity) objectively (i.e., non-dependent on the analyst). This issue conditions the appropriateness in the experimental use of referential frameworks of cut marks made on defleshed bones. It could also affect the potential inference of hominin access to carcasses by providing support for butchery of fleshed or defleshed carcasses in prehistoric contexts. This comparison of such structurally similar marks was adopted here on purpose with the intention of challenging the recently discovered exceptional capability of AI tools to provide highly accurate classifications of BSM^1^. By keeping the effectors the same as well as the bone types in both experiment types, only contextual differences in the presence/absence of meat would condition any potential difference of marks that *a priori* should be structurally identical. Given the challenge posed by this experiment in identifying and discriminating cut marks produced in both contexts, these types of marks were selected in the present work to test the efficiency of DL methods in BSM classification.

The use of DCNN in BSM analysis was the first study to use a completely objective method in the assessment and identification of BSM, based on an artificial intelligence approach^1^. Given that no other method has differentiated structurally-similar BSM (cut and trampling marks) as accurately as DCNN, we will use a similar AI approach here on an experimental set of cut marks made on defleshed bones and on fleshed bones butchered with stone tools. The hypothesis to be tested here is whether the diversity of cut mark morphologies created by the types of tool-bone contacts (on fleshed/defleshed elements) handicaps any useful distinction of cut marks between experimental scenarios, especially since the tool kit used was the same for both experiments. Also, the number of cut marks made with these tools varied since in the butchered sample, cut mark production could not be as controlled as on the defleshed bone sample. We predict that if these variables are any influential in cut mark morphology, we should expect divergent morphologies in cut marks from both experiments and an accuracy rate much higher than random guessing. The alternative is that these variables are not influential in the overall microscopic features of cut marks and that BSM made in either experimental conditions cannot be differentiated.

## Method

### DL architecture

CNN are usually composed of a sequential series of artificial neuron layers (multilayer perceptrons) that receive input information and produces output information through a transformation process involving weights, biases and activation functions in each layer^12^. Weighted inputs are pushed through an activation function that determines the threshold of neural activation and its signal. This signal travels downward through the multiple layers (deep learning refers to having multiple hidden layers) and emerges in the last output layer, whose activation function is determined by the type of classification problem. The resulting output is then compared to the expected output from controlled classification and the error is backpropagated sequentially through each layer (in inverse order) updating the weights in each layer (and each neuron) in proportion to their contribution to the bias. This constitutes the learning process of the training neural networks (NN)^13–15^. During backpropagation, momentum (direction of weight modification even when error decreases) and learning rate decay (artificial decrease of learning rate to avoid overfitting and enable increasing predictive properties) can be implemented. Each complete backpropagation constitutes an epoch. Multiple epochs increase the learning rate of the algorithm by correcting the sequentially smaller errors.

Sequential layers are created with a starting convolutional layer. It applies a convolutional process to the input, consisting of computing the dot product of the weights of the layer and a small region they are connected to in the input layer^16^. This layer (i.e., batch area) is a receptive field. This field has predetermined dimensions and slides a certain number of steps on the layer over a determined number of small areas creating a feature map^17^. It is this feature map that detects specific features on images. Pooling layers use feature maps to compress information and generalize features, which reduces the overfitting of the training data. Pooling layers also have a receptive field (usually, smaller than those of convolutional layers). Pooling features generates a second feature map. This alternation between convolutional and pooling layers end with the final fully connected layer. This flat feedforward neural layer uses a nonlinear activation function, which depends on the classification problem at hand (e.g., “softmax” for multiple categories or “sigmoid” for binary classification).

After training, the NN must be tested against a testing or verification sample. This yields the accuracy rate of a NN and its predictive and classificatory potential. The three most crucial elements of the training network are the loss function, the optimizer and the metric selected for monitoring the functioning of the network. The loss function measures how the network is performing on the training data. The optimizer is an algorithm that updates the network according to the loss function. For example, Stochastic Gradient Descent (SGD) is one of the most commonly used optimizer for training neural networks^18,19^. The reason is that it is fast and comprehensive. Batch methods based on complete training samples tend to converge to local optima producing overfitting and poor predictive/classificatory results on testing samples. Computing the gradient and cost on full training sets can be cumbersome. This is where SGD and other similar algorithms (e.g., Adam) constitute an improvement. Instead of running backpropagation on the complete training set, SGD allows to select just a portion of it randomly and reach full convergence rapidly. Finally, the metric monitors the network performance on the training and testing data sets. Usually “accuracy” is the most selected metric type^14^.

Here, a DCNN model was used to classify images of cut marks made on fleshed and defleshed bones (see below for description of samples). All images were transformed into black and white, by using bidimensional matrices, and they were reshaped to the same dimensions (80 × 400 pixels). The Keras library was used with the TensorFlow backend. Computation was carried out on a GPU HP Workstation. The CNN model selected was sequential. The model architecture is shown in Table 1. The model conisted of a first convolutional layer composed of 32 nodes (with a kernel of 3 × 3), followed by another one with 64 nodes and two consecutive ones with 128 nodes. In between, pooling layers (with a kernel of 2 × 2) were used. The model was flattened with a dense layer of 512 nodes. This is a modification of the basic structure of the more complex VGG16 architecture. The activation function for each layer was a rectified linear unit (ReLU). Both this function and its combinations are non linear. Because of sparse activation (fewer neurons are activated) the network is lighter and progresses more efficiently. The fully connected layer of the network used a “sigmoid” activation, which is a logistic activation with a probability distribution of the different binary classes. The loss function selected was binary cross -entropy given that the test was based on a binary classification problem and the output depends on probabilities. Cross-entropy measures distances between probability distributions and predictions^14^. The “RMSprop” optimizer was selected. RMSprop is similar to Gradient Descent with momentum. The differences lies in the procedure of calculation of gradients. By limiting vertical oscillations, RMSprop increases the learning rate and converges faster. Accuracy was the metric selected for the compilation process.

**Table 1:** Neural network model and parameters used for the present study (simple model).

Layer (type)	Output Shape	Param #
conv2d_1 (Conv2D)	(None, 78, 398, 32)	896
max_pooling2d_1(MaxPooling2	(None, 39, 199, 32)	0
conv2d_2 (Conv2D)	(None, 37, 197, 64)	18496
max_pooling2d_2 (MaxPooling2	(None, 18, 98, 64)	0
conv2d_3 (Conv2D)	(None, 16, 96, 128)	73856
max_pooling2d_3 (MaxPooling2	(None, 8, 48, 128)	0
conv2d_4 (Conv2D)	(None, 6, 46, 128)	147584
max_pooling2d_4 (MaxPooling2	(None, 3, 23, 128)	0
flatten_1 (Flatten)	(None, 8832)	0
dense_1 (Dense)	(None, 512)	4522496
dense_2 (Dense)	(None, 1)	513

Total params: 4,763,841.

Trainable params: 4,763,841.

Non-trainable params: 0.

Training under supervised learning was performed on 70% of the original sample. After training the algorithm, validation and testing was carried out on the remaining 30% of the sample. Training and testing were performed through mini-batch kernels (size = 20). For training, a total of 100 epochs were used and 100 steps per epoch were selected. In the controlled experiment carried out with a subsample (see below), batch size was 32 for the training sample and 10 for the validation/testing sample. Each epoch was trained by a number of steps porportional to sample size.

We must emphasize that we intentionally kept the model simple, within certain parameters. Usually, different models are compared and tuned with parametrization, which involves comparing accuracy in models that become increasingly complex. Complexity aims at minimizing overfitting of the training sample and maximizing accuracy of testing samples. Here, we did not apply weight regularization (i.e., constraining the network to take small weights) or dropout (randomly discarding output features of a layer) to avoid overfitting^20^. We did this intentionally after observing the high accuracy on the testing sets obtained by a simple architecture (Table 1). This was quite unexpected because human experts are not able to make that discrimination. We had anticipated that the machine would also show that both types of cut marks (on fleshed and defleshed bones) were undifferentiable. However, we tested for overfitting by comparing learning curves from training and validation sample subsets. Both the low- and intermediate-resolution data sets were made with hundreds of cut mark images (see below). The high-resolution was restricted to fewer images. For this reason, for this data set we proceeded by implementing image augmentation, as a way to prevent overfitting, as recommended in standard protocols^14^. The sample was augmented via random transformations of the original images involving shifts in width and height (20%), in shear and zoom range (20%), and also including horizontal flipping and a rotation range of 40°. This implies that multiple different images are obtained from each of the images of the original data set, thus increasing exponentially the sample size used by the algorithm. Data augmentation was also used for the controlled experiment carried out with the intermediate-resolution data set (see below).

The high-resolution data set was also challenged by analyzing first both image data sets with their most immediate cortical surface and then carrying out a subsequent analysis only in the internal part of the groove of each mark for both data sets. This latter approach was comparing marks that were so similar that the model architecture became too simple and showed a decreased (yet substantially high) accuracy. In this case, we used a dual model approach (simple and complex). The use of a complex model was intended to capture the microscopic features inside the grooves better than the simple model. It was also used with the goal of further reducing the impact of bias/variance effects by introducing more regularization methods. The selected model was Alexnet^12^. This model was selected over other similar ones, because of its fast computation. Alexnet consists of a sequential model of eight layers. The first five layers contain CNN followed by max-pooling layers topped by three successive fully connected layers. It has 96 kernels of size 11 × 11 × 3. The pooling layers contain 3 × 3 filters with a stride of 2. The activation function for each layer was a rectified linear unit (ReLU). The last fully connected layer of the network used a “sigmoid” activation, as in the previous model. The loss function selected was binary cross entropy. The “RMSprop” optimizer was selected. Accuracy was the metric selected for the compilation process. The model was used with data augmentation to reduce overfitting. The authors increased the original sample that they used by a factor >2000 using this approach. The model also contains three “Dropout” layers. Dropout is a regularization method that consists of random switching off of neurons, which do not participate in the backpropagation process. This produces that every epoch uses a different network architecture, thus making the model more flexible to learning and less prone to overfit. To make computation more efficient and to contribute to minimizing overfitting, the minibatch method was also used here. Batch size was five images per batch. Here, we applied it also in conjunction with data augmentation and run it for 50 epochs. The full model is displayed in Table 2.

**Table 2:** Summary of the architecture of the Alexnet model.

Layer (type)	Output Shape	Param #
conv2d_1 (Conv2D)	(None, 18, 98, 96)	23424
activation_1 (Activation)	(None, 18, 98, 96)	0
max_pooling2d_1 (MaxPooling2	(None, 9, 49, 96)	0
batch_normalization_1 (Batch	(None, 9, 49, 96)	384
xconv2d_2 (Conv2D)	(None, 1, 41, 256)	1990912
activation_2 (Activation)	(None, 1, 41, 256)	0
max_pooling2d_2 (MaxPooling2	(None, 1, 21, 256)	0
batch_normalization_2 (Batch	(None, 1, 21, 256)	1024
conv2d_3 (Conv2D)	(None, 1, 21, 384)	98688
activation_3 (Activation)	(None, 1, 21, 384)	0
batch_normalization_3 (Batch	(None, 1, 21, 384)	1536
conv2d_4 (Conv2D)	(None, 1, 21, 384)	147840
activation_4 (Activation)	(None, 1, 21, 384)	0
batch_normalization_4 (Batch	(None, 1, 21, 384)	1536
conv2d_5 (Conv2D)	(None, 1, 21, 256)	98560
activation_5 (Activation)	(None, 1, 21, 256)	0
max_pooling2d_3 (MaxPooling2	(None, 1, 11, 256)	0
batch_normalization_5 (Batch	(None, 1, 11, 256)	1024
flatten_1 (Flatten)	(None, 2816)	0
dense_1 (Dense)	(None, 4096)	11538432
activation_6 (Activation)	(None, 4096)	0
dropout_1 (Dropout)	(None, 4096)	0
batch_normalization_6 (Batch	(None, 4096)	16384
dense_2 (Dense)	(None, 4096)	16781312
activation_7 (Activation)	(None, 4096)	0
dropout_2 (Dropout)	(None, 4096)	0
batch_normalization_7	(Batch(None, 4096)	16384
dense_3 (Dense)	(None, 1000)	4097000
activation_8 (Activation)	(None, 1000)	0
dropout_3 (Dropout)	(None, 1000)	0
batch_normalization_8	(Batch (None, 1000)	4000
dense_4 (Dense)	(None, 1)	1001
activation_9 (Activation)	(None, 1)	0

Total params: 34,819,441.

Trainable params: 34,798,305.

Non-trainable params: 21,136.

In case of a high accuracy, a gradient visualization for detecting the features that influenced discrimination was applied using a gradient weighted activation mapping algorithm (Grad-CAM)^21,22^. This method overlays a heatmap on the original image based on gradients of the predicted class derived from the last convolutional feature map. The Grad-CAM algorithm highlights areas of the marks that are most important for the prediction and classification of the image. The use of this algorithm showed the importance of certain areas for correct image classification.

### Sample

The null hypothesis of this study is that cut marks made with stone tools on defleshed bones and those resulting from tools used during butchery of fleshed carcasses do not differ in their microscopic attributes. Given that we intended to reproduced a large portion of the diversity of morphologies caused by diverse properties of tool types and raw material types, we used a large array of tools and three types of raw materials (Supplementary Table S1). This would provide more information to the machine and potentially improve its training process. It should be emphasized that the same tools were used for both experiments. The experiment on defleshed bone was conducted first with the aim of limiting the number of imparted cut marks by tool to a maximum of 20 and, thus, ensuring that tool edges underwent no damage or modification. Bones from both experiments were cleaned equally (see below). All methods were performed in accordance with the relevant guidelines and regulations. Bones and fleshed carcass parts were obtained from a commercial butcher, which complies with regulations according to the Spanish Ministry of Health. The experiments were conducted following the approval and protocols implemented in the Institute of Evolution in Africa.

The experiment was designed in three stages, from lower to higher resolution. Low resolution implied that the experimental contingency was substantial given the large number and diversity of effectors used (Supplementary Table S1). Two experimental sets tested microscopic features on a BSM sample created on defleshed bones (1087 cut marks) and another one resulting from bulk defleshing (203 cut marks). Marks were imparted on the same element types (bovid humeri, femora, radii and tibiae) and bone portions (midshafts) on both experiments to avoid any potential bias introduced by heterogeneous combinations of bone portions and element types. Marks on the fleshed and defleshed bone samples were made with the same 54 stone tools (37 retouched flakes and 17 handaxes) (Supplementary Fig. 1S). Marks on the defleshed bone sample were more abundant because they were initially created with the idea of using them in a separate study. This large sample was used here to test the hypothesis of marks being identical regardless of context (bones with/without meat). Given that both samples contained uneven numbers of marks made with the same tool types, some discrepancies in the resulting morphologies of marks were expected, probably yielding higher discriminatory rates than a sample derived from a more homogeneous tool set. Slight inconspicuous modification of the tool edges could also have played a role in making different marks in the second experiment, although we did not macroscopically appreciated any change. Heterogeneity was further introduced by the combination of three different raw materials in the tool kit (flint, quartzite, sandstone). The hypothesis for this experimental set was that it would yield the highest accuracy rates in the classification of marks, given the heterogeneity of the tool set and the variable number of resulting marks made with each tool. Here, higher accuracy was defined as a percentage of correct classification significantly above the probabilistic threshold of 50% expected from random guessing.

The intermediate-resolution experiment consisted of using only a subset of cut marks resulting from the use of the same five retouched flakes that were utilized both on defleshed and fleshed bones, producing a total of 132 cut marks on the fleshed bone sample and 101 cut marks on the defleshed bone sample. These five flakes were selected because they were microscopically observed to have preserved their edges intact after their use on the defleshed bones (Supplementary Fig. 2S). This made them appropriate for the experiments because differences of cut marks morphologies could not be attributed to sharp *versus* dull edges. Here, the hypothesis was that mark identification accuracy would be lower than in the previous experiment, because marks were created only with one tool type (retouched flakes) and all tools were the same for both experiments. However, even here it could be argued that since the same five flakes were used for cutmarking defleshed bone, their subsequent use on fleshed bone could already have biased the sample by having their edges been microscopically modified during their previous use on defleshed bone surfaces. To test this hypothesis, we performed a control experiment with this intermediate-resolution sample. We used an additional sample of cut marks on fleshed bone made with six new flakes that were very similar in size to the five original flakes. Marks were created sequentially on the fleshed bone (from middle to one end). To prevent any possible effect of edge metamorphosis during butchery, we limited the analysis to the first ten marks per each flake preserved on the bone surface. This subsample was then tested against the intermediate-resolution sample to check for statistical differences, which if existing, should be explained by different degrees of edge dullness and not by any other biasing factor. Before carrying out this experiment, we performed within-group random analyses to detect if the cut marks made with the same flake were different in the same bone and experiment type and by permuting random numbers of marks made with the same flake on bones from both experiments. All DCNN models yielded negative results with accuracy rates <50%, showing no discrimination beyond random guessing. This indicated that the edges of the flakes had not undergone any significant modification in between experiments to be reflected in microscopically-different types of resulting cut marks.

The high-resolution experiment consisted of 48 cut marks made on defleshed and fleshed bones using in all cases the same single flake (Supplementary Fig. S3). High-resolution was defined as higher control on mark variability given the use of a single effector. Here, given that the tool was the same for both experiments, a lower accuracy in the classification of marks was expected if the contextual part (i.e., presence/absence of meat) of the analogy did not play any role^23^. Failure to justify this hypothesis could not be attributed to the same tool having its edge modified by use from one experiment to the other, because it was checked for sharpness prior to having been used in both experiments. As an additional comparison of the DL method used in this study and previous multivariate analytical approaches to BSM classification^24^, this subsample was also analyzed following the same protocol as described by Domínguez-Rodrigo *et al*.^24^ using a multivariate set of microscopic variables and its results were compared to those from the present analysis. A logistic regression (LR) analysis was used for this purpose. This LR was made by dividing the sample into training (70%) and testing (30%) subsamples. Sample partitioning was made using a function involving proportionate class random allocation of cases to the training and testing subsamples. Training was made using a general linear model based on a binomial logit regression. Given the small sample size, the selected sample was trained with a control method involving repeated k-fold cross-validation. The regression was cross-validated using k = 5. The analysis was made using the R “caret” library.

Bones were cleaned with a solution of neutral detergent and boiling water. The cleaning process was the same for both experimental scenarios in each of the three types of experiments (low to high resolution). Flesh did not bias the cleaning on the fleshed bone sample because bones were utterly defleshed prior to being boiled, since we performed complete butchery on those bones. After scrutinizing marks from both experimental sets, we documented the presence of all the inconspicuos microscopic features that are easily modified when biostratinomic processes operate, such as microstriations, shoulder effects and internal and external flaking. This suggested that the cleaning process was not a biasing factor in both samples. However, since cleaning through boling might have a potential biasing effect and no previous research has documented its effect on BSM micromorphology, we carried out an additional control experiment, consisting of cut marks made on a set of already clean bones, which were artificially wrapped with meat with a thickness of one inch approximately. The hypothesis was that if boiling had any distorting effect on marks, the controlled sample would differ micro-morphologically (and also statistically) from the other two samples made on boiled bone, but more specifically from the sample of marks made on fleshed bones, because they took twice as much time to boil to degrease them properly. A second version of this hypothesis was that if the control sample differed from the defleshed bone sample but not from the fleshed bone sample, cleaning should be ruled out as a biasing factor and, the alternative hypothesis that presence or absence of meat determines micro-morphological characteristics of cut marks would be confirmed.

Marks were then documented with a binocular microscope (Optika) at 30 X and images were taken in this magnification. The resulting image data bank (composed of 1290 cut marks from the three-stage experiments plus 62 additional cut marks from the control experiment) was used for analysis through the DCNN model described above. Marks were uploaded with the surrounding cortical surface into the machine. After detecting that a few images were potentially biasing the results by incorporating features outside the marks, images for the high-resolution experiment were trimmed to the extreme of showing only the shoulder boundaries and the inside of the groove. It was only in this subset that only cut mark features were used by the algorithm to provide accurate classification of all marks of both experimental scenarios.

### Ethical approval

The methods were carried out *in accordance with* the relevant guidelines and regulations.

## Results

**Figure 1:**
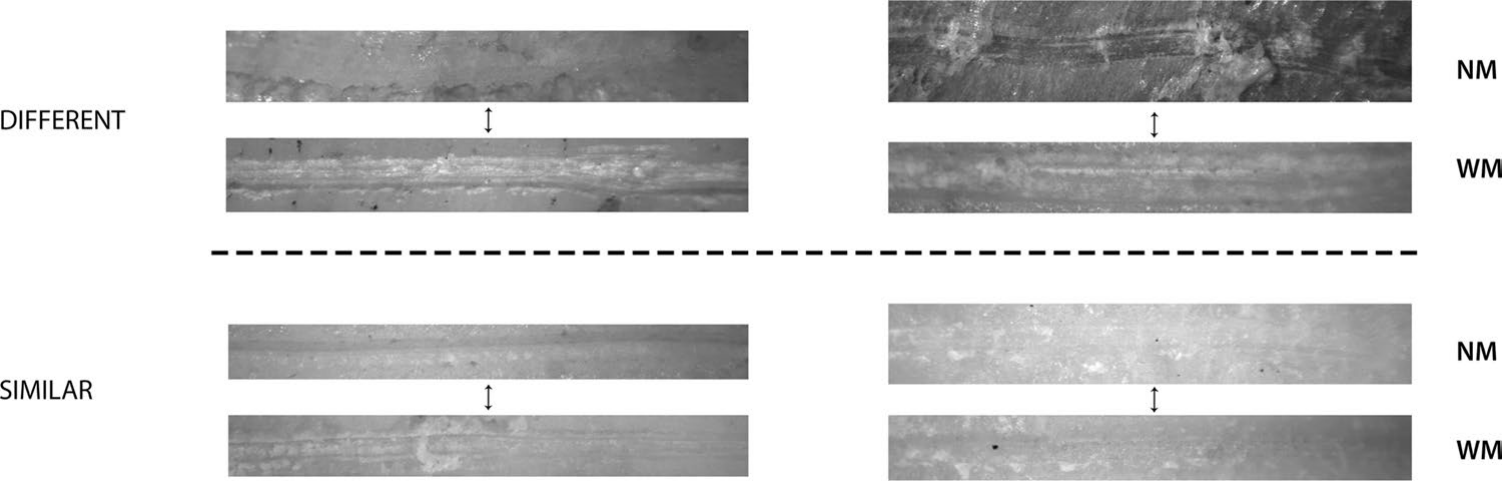
Selection of marks from both experiments showing overlapping similarities and contrasting differences. The upper half shows contrasting images of cut marks made on bones with meat (WM) and no meat (NM). The lower half shows images of visually indifferentiable cut marks made on bones with and without meat.

For the low-resolution experiment, the testing sample yielded an accuracy of 94.53% of correct classification of cut marks for both experiments. A moderate accuracy was expected because of the heterogeneity of tools used in both experiments. However, this high degree of accuracy was not anticipated and, it certainly indicates that at the microscopic level cut marks made on fleshed and defleshed bones are differentiable (Fig. 1).

**Figure 2:**
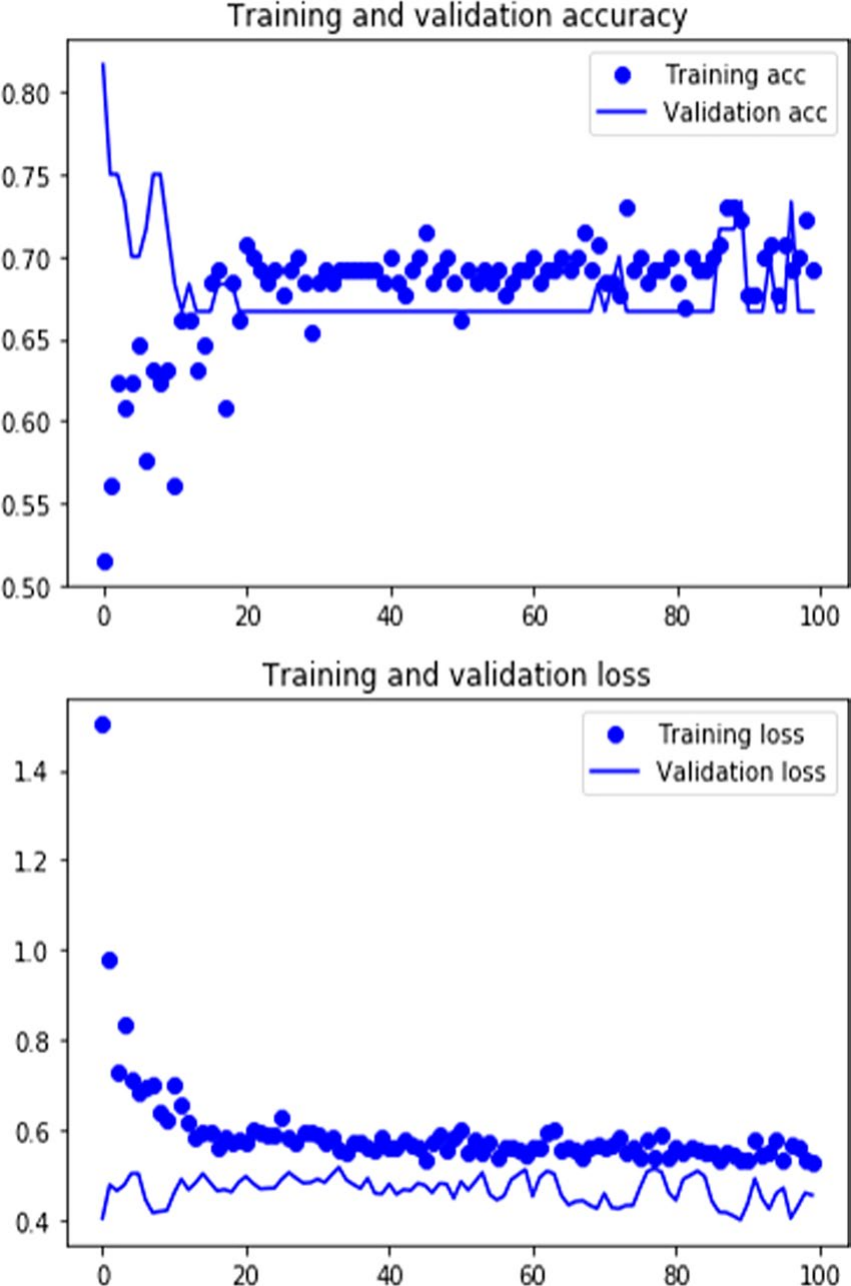
Loss (y-axis) and epochs (x-axis) (**lower**) and accuracy (y-axis) and epochs (x-axis) (**upper**) of the control experiment comparing cut maks on fleshed bone made with the same five flakes that were used for imparting marks on defleshed bone and the new control flakes.

For the intermediate-resolution experiment, when comparing both samples, the testing samples yielded an accuracy of 95.54% in the identification of cut marks made on fleshed and defleshed bones by the same five retouched flakes. This result was unexpected and goes against the hypothesis of similar cut marks resulting from both experimental scenarios, given that the tools used were the same. To test the hypotheses that edge dullness may have biased the experiment (since the same five flakes were used for both samples) or that the cleaning process may have impacted the final configuration of marks on both experimental sets, since they were boiled for different times, we used the control side experiment on clean bone and with fresh flakes. When compared with the cut mark subset from the defleshed bone sample, the DCNN model classified successfully 76% of marks from both sets. An exact binomial test, testing the null hypothesis about the success probability in a Bernoulli experiment yielded a significant probability of random guessing of 58.8% (with a 95% confidence interval of 0.33–0.67%). The DCNN result clearly shows that the high accuracy rate separating both mark sets is not random. When the control sample was compared to the subsample of cut marks from fleshed bone, it yielded an accuracy of 66.6%. This is not outside the range for random guessing. The same model that classifies cut marks between the fleshed and defleshed bone samples so successfully seems to be unable to learn how to discriminate when comparing cut marks from the control and the fleshed bone samples. This is documented not only on the validation set but also even in the training set (Fig. 2). This further shows the similarity of the microscopic features of both sets of cut marks.

For the high-resolution experiment, the testing samples yielded an accuracy of 88.89% in the identification of testing cut marks made on fleshed and defleshed bones by the same single effector. This result was unexpected and goes against the null hypothesis of similar cut marks resulting from both experimental scenarios, given that the single tool used was the same in both cases. The combination of these three-stage resolution experiments showed that when the structural and substantial parts of the analogy were held constant, only the contextual/environmental part of the analogy was influencing the results, as stressed by Bunge^23^.

Given that the results obtained were against the null hypothesis, several alternative tests to search for possible factors conditioning the result were made using the same DCNN model. For example, just in case other unknown factors were playing a major role in this reported high accuracy, we selected one experimental sample (marks made on fleshed bones) and artificially divided it up into two separate groups, labelled A and B and then analyzed it with the same DCNN model. The result of 58% of correct classification did not differ statistically from random guessing. Therefore, the algorithm was working perfectly and knew very well when cut marks belonged to the fleshed-bone and defleshed-bone experiments.

The LR analysis of the 48 marks used for the high-resolution models were analysed using the same protocol as Domínguez-Rodrigo *et al*.^24^ (Table S1). The LR yielded a low level of classification showing that both types of marks were similar. The accuracy produced by this analysis was 64%; only slightly (and non-significantly) higher than the average probability expected from random guessing. None of the variables showed any significant input into the classificatory potential of the test. All variables showed p-values of 0.99, except “shoulder” (p = 0.24). Sensitivity (66%) and specificity (62%) were very similar. As indicated above, for the same type of images, the DCNN model yielded a much higher accuracy (88.9%). Does this mean that computer vision exceeds any other method at BSM classification? Does it mean that cut marks made with the same tool in both types of experimental scenarios are conditioned by the “context” (i.e., bones with meat or no meat)? From this information, an initial working hypothesis is that marks made on a defleshed bone would probably generate more groove shoulder modifications than when made on fleshed bone. Flesh may buffer the contact of flake edge periphery and cortical bone surface in a better way than when made on defleshed bone. The LG data would suggest that DCNN are using BSM shoulders as discriminatory features. To approach this hypothesis, it must be known what features the DCNN algorithm perceives in marks to classify them so successfully.

**Figure 3:**
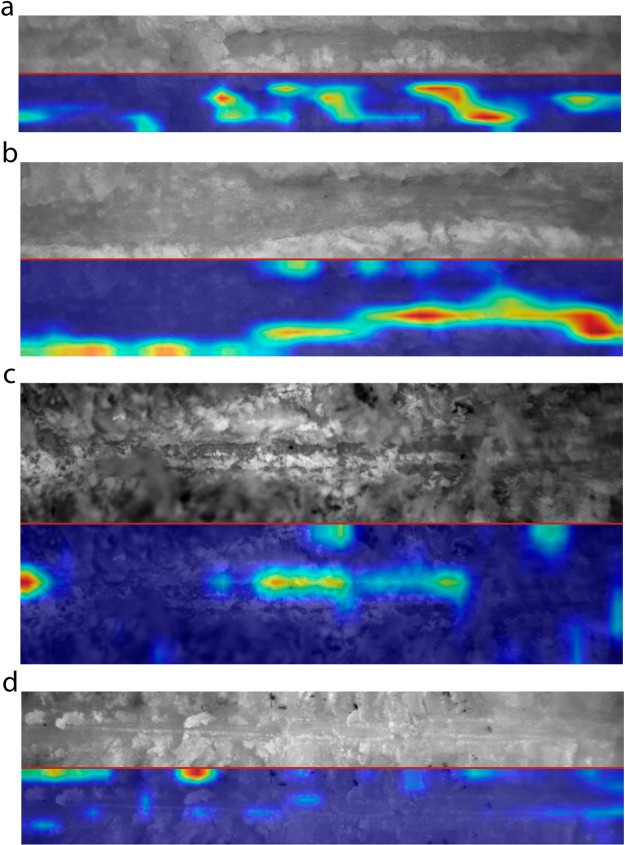
Heat maps produced by the Grad CAM algorithm indicating discrimination areas important for correct classification of the complete image data set (obtained from the low-resolution experimental subsample). (**a**,**b**), Cut marks made on bones with meat. (**c**,**d**) cut marks made on defleshed bones. Red hat only show in the heat map denotes the most important features.

The Grad-CAM algorithm highlighted that the shoulders of some cut marks on defleshed bones were important for classification (see location of the red gradients on Fig. 3a). However, many cut marks made on defleshed bones were observed to exhibit little or no shoulder and flaking. In those cases, the DCNN still succeeded in classifying most of these marks correctly. In such cases, the Grad-CAM algorithm highlighted very inconspicuous features related to the interruption of marks by internal flaking or disappearance of the microstriation trajectories (Fig. 3c,d), which occurred more frequently on the defleshed bone sample. Lastly, given the powerful discriminatory potential of the algorithm, when marks were so similar that separating them by their internal features became impossible for the DCNN model, it was the presence of inconspicuous features on the peripheral cortical area adjacent to the shoulder that determined the ascription of the BSM to a class (Fig. 3c,d). How those stochastic microscopic features were successfully discriminated by the model remains unexplained. This emphasizes that image capture of BSM must be made as close to the groove as possible, to minimize the effect of other existing microscopic features, not necessary related to the intrinsic structural characteristics of BSM.

For this reason, to test the hypothesis of whether the internal features of cut marks were the same in both experimental scenarios, and to avoid any interference of any microscopic feature documented outside the marks, the original images of the high-resolution experiment were trimmed at the shoulders and only the shoulder edges and the interior of the marks of both experiments were compared. The DCNN model was run again and in this case the accuracy became slightly lower than when using the complete image (77.78%). We suspected that would be the case because when scrutinizing just the internal part of the groove, some important shoulder and near-shoulder modifications are lost and accuracy should be impacted by a greater similarity of the internal miscroscopic features. However, another important factor is that the model architecture is too simple for such similar data sets. For this reason, we re-run the analysis with the complex model (Alexnet) (see above, Table 2). This model captured the internal features of marks better than the simple model and reduced overfitting. The result was a 88.8% of correct classification of marks made on fleshed and defleshed bones. This model reproduced the accuracy of the test run on non-cropped marks with the same exactitude because of the limited testing sample. With almost 90% of accuracy, the high-resolution small data set compares well with the 95% of accuracy achieved by the low- and intermediate resolution data sets. Although the interpretations of the high-resolution data set are limited by sample size, they coincide with those resulting from the other two more extensive data sets, supporting (against our initial expectations) that cut marks made on fleshed and defleshed bone can be discriminated in a high proportion.

In the high-resolution experiment, a matrix derived from the one hot encode dataframe of pixels was compared in between experiments and differences were shown to be significant (compared to random guessing) in the model with mark shoulders and their surrounding periphery (88.89%, p = < 0.05), as well as in the second model involving only the internal part of the grooves (77.78/88.8%; p = < 0.05).

**Figure 4:**
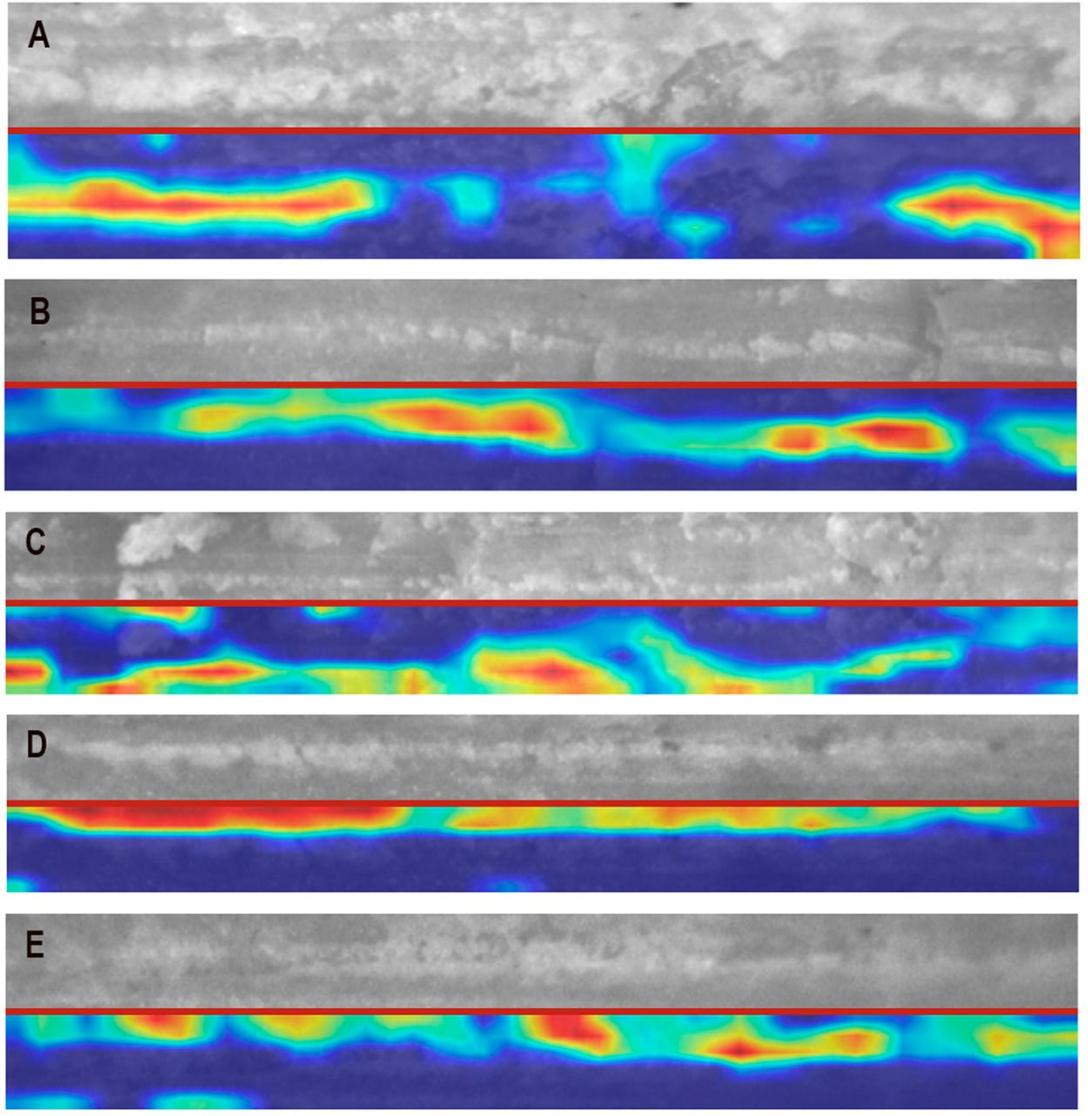
Heat maps produced by the Grad CAM algorithm indicating discrimination areas important for correct classification of the high-resolution cropped images showing only the mark grooves. (**A**–**C**) Cut marks made on defleshed bones. (**D-E**), cut marks made on bones with meat. Red color in the heat map denotes the most important features.

The Grad-CAM algorithm highlighted special areas inside these marks as selective for attributing them to class. It was clear that the algorithm was not specifically selecting only the mark shoulder as suggested by the LG analysis as the most discriminatory part; probably because similar types of shoulder flaking occurred in both experiments. Given the overall identity of the structural features of cut marks in both experiments, it seems that the DCNN model was forced to select very inconspicuous features as discriminatory; among these, it was repeatedly found that the more frequent flaking occurring inside the groove in cut marks made on defleshed bone was a crucial classificatory feature. This occurs in much lower frequency in cut marks made on fleshed bone (Fig. 4). Images classified as cut marks from fleshed bones also showed a higher number of disruptions of the trajectory of features inside the grooves, which were selected as discriminatory by the algorithm. To be sure that most of the features that the model was selecting belonged inside the groove, we carried out the same experiment masking the mark grooves and exposing the rest of the image to the algorithm. The DCNN model was unable to classify both sets of BSM beyond random expectations.

In sum, the sequence of experiments demonstrate that the null hypothesis of equal microscopic mark features in both experiments must be rejected. Overall, cut marks made on fleshed and defleshed bone look very similar, but the higher frequency of flaking and microstriations documented in cut marks made directly on defleshed bone seems to be conspicuous enough to enable high accuracy in BSM classification by the DCNN model.

## Discussion

The results from the control experiment when compared to the intermediate-resolution samples suggest that: a) the mark sets from the control sample and the fleshed sample can be only slightly differentiated, but not statistically since differences can be random; b) the slightly higher accuracy than the theoretically expected average probability of random guessing in the control sample could also reflect the impact of having used different tools for both sets or the bias introduced by boiling; c) tool dullness did not condition the outcome, since only fresh flakes were used in the control experiment (which indirectly shows that the flakes used for the intermediate-resolution fleshed bone set were in good condition) and, d) boiling might, after all, have some minor (yet, hard to detect) effect, but such purported effect seems to have been minor compared to the variable presence/absence of meat on the bone. This is shown by the control set being much more similar to (and statistically undifferentiated from) the fleshed bone mark set, which had boiled more than twice the time used for cleaning the defleshed bone cut mark sample. Given the substantially (and statistically significant) differences in successfully discriminating cut marks from the control and the defleshed bone sample, it can be argued that presence of meat plays an important role in the micro-configuration of cut marks. The reported differences may be caused by meat buffering the abrasive effect of tool edge and bone surface, thereby reducing the presence of microstriations, shoulder effect and flaking.

The contrast between the low accuracy of the LR (64%) and the high accuracy of the high-resolution experiment using DCNN (88.89% from complete images and internal groove images) supports the high accuracy obtained in low- and moderate-resolution experiments (95%) and shows that the discrimination is consistently linked with the heuristic provided by different analytical methods. Potential equifinality, as inferred from the use of LR, can be overcome with the use of DCNN. This reinforces von Bertalanffy´s definition of equifinality as only a temporary state^25^. This also shows the superior power of DL methods for correct identification of bone surface modifications over traditional ones. Not only do they yield a significantly higher accuracy in classification, but they also provide unambiguous objective criteria for mark identification, thus avoiding the potential bias introduced by the analyst^9^. A documented observation of traditional multivariate methods was that categorization of variables involved a high degree of subjectivity by the analyst^9^. This conditioned the data obtained and their subsequent interpretation, with different authors tallying variables differently and reaching divergent interpretations. It was emphasized that only objective methods could bring a more scientific approach to BSM studies^9^. This was initially achieved by the pioneering use of DL methods for the discrimination of cut and trampling marks^1^. In that study, it was shown how the collection of information could be automated and completely objective. It was also shown that cut mark shoulders played a major role in the high accuracy achieved. Cut marks and trampling marks showed different areas for discrimination on a systematic basis. Here, this observation has been reinforced. Traditional multivariate methods document the presence of shoulder-affecting processes, but given their categorical nature, they are unable to capture their extent and input into the classification process. Shoulder flaking occurs on cut marks created while bulk defleshing, but it seems less developed than in marks resulting from cutting on defleshed bone, as shown here (Fig. 2). However, given its variable presence in both experimental sets, it is the more inconspicuous flaking inside the groove that discriminates cut marks from both experiments best (Fig. 3). The DCNN feature maps generated by the convolutional layers identify this difference in the magnitude of flaking much better.

Does this mean that cut marks resulting from bulk defleshing and those imparted on defleshed bones are structurally different? The answer is negative when one considers the complete set of features inside of the marks used in previous methods^24^. LR models show that inside the groove, both types of cut marks are very similar when considering most microscopic features. This is why traditional multivariate approaches fail to reach any significant degree of accuracy. It is only with flaking (both on the shoulder and, specially, inside the groove) that discrimination becomes apparent.

It remains to be tested whether the lower degree of modification of marks made during butchery of fleshed carcasses can be differentiated from other structurally-similar modifications, such as trampling marks, also characterized for having their shoulders and internal walls either unmodified or modified only slightly^24^. Given that traditional methods discriminated successfully with data from samples derived from bulk defleshing^24^, the null hypothesis is that cut marks from butchery will still show a higher degree of shoulder and groove modification than trampling marks and accuracy in classification will be high when comparing cut marks made with retouched flakes and trampling. Accuracy should be even higher when comparing trampling with cut marks made with simple flakes given that groove shape and internal microstriations differ even more^24^.

An additional experiment is needed to ascertain whether cut marks stemming from bulk defleshing can also be differentiated from trampling marks after the former have been modified by diagenesis or by biostratinomic factors affecting cortical preservation and, especially, shoulder modification^5,6^. Gaudzinski *et al*.^4^ insisted that experimental cut marks could not be reliable proxies for archaeological cut marks exposed to continuous modification by dynamic biostratinomic (i.e., trampling) modifications. Further experiments should model this morphing of pristine cut marks during their taphonomic evolution. The null hypothesis is that cut marks from “static” experiments and those from “dynamic” longitudinal experiments will be successfully differentiated by DCNN models. An additional null hypothesis is that cut marks from “dynamic” experiments and trampling marks will also be successfully differentiated. We hope the present work encourages the testing of this new set of hypotheses with future experimentation. In the end, the image libraries obtained with these types of experiments will constitute the foundation for effective identification of cut marks in the archaeological record subjected to different degrees of modifications by their taphonomic evolution using artificial intelligence tools.

An additional concern about the use of this actualistic analog is that the features that characterize these cut marks are completely preserved in the modern experimental samples but they may have disappeared from fossil marks. Even under the assumption of absence of modification by biostratinomic factors, diagenesis could be a biasing process after burial due to soil chemistry. This questions that archaeological cut marks could be as efficiently identified by this and other methods as modern experimental marks are. There is no doubt that this is a conditioning factor in reliably identifying cut marks from the archaeological record. However, experimental work has shown that it takes very little to alter some of the most diagnostic microscopic features of cut marks (ie., microstriations, shoulder effect and flaking). In cases where these features have been modified by biostratinomy or diagenesis, there is little hope is objectively recognizing cut marks. In contrast, in cases where archaeological marks display these microscopic features, there is a potential that these marks will be as confidently identifiable as modern experimental cut marks. There are indeed cases in the archaeological record where these features are as pristinely preserved as in modern experiments. We find them in several Olduvai sites, for example, where the bone preservation conditions were exceptional. Therefore, we caution against using this experimental referential frameworks on marks that have been substantially biased by biostratinomic and diagenetic taphonomic processes but encourage its use on well-preserved assemblages.

The archaeological implications of this method and its results are important. If cut marks made on fleshed and defleshed bones are different, maybe BSM inferred to be caused by access to largely defleshed carcasses by hominins could be successfully differentiated in archaeofaunal assemblages from those made by bulk defleshing. This would add more heuristics to contrasting interpretations of early hominin hunting or scavenging behaviors. This discovery, methodologically, is also of utmost importance. It provides a cautionary note on the use of diagnostic criteria of cutmarks derived from experiments where marks were made on defleshed bones.

## Conclusions

DL methods, through DCNN models, have successfully differentiated cut marks made on fleshed and defleshed bones based on subtle contextual differences. These differences relate mostly to the presence of microstriations and the extent of flaking and cortical crushing in both experimental cases. This is of utmost taphonomic relevance, because it underscores that: (a) the internal morphology of cut marks although similar, differs microscopically in both experimental samples; contra previous suggestions^26^, (b) the difference is mostly related to the extent of microstriations and flaking rather than presence/absence of within-groove modifications and, (c) these features barely survives time^27^, since they are very inconspicuous or non-existent in several archaeological cut marks, rendering cut marks during butchery a better proxy of archaeological cut marks.

The present work questions the validity of the experimental cut marks made with handaxes using defleshed bones when shoulder modification plays a crucial role in discrimination^26^. It is our opinion that cut marks with handaxes from previous experiments should be compared again with marks made with simple and retouched flakes using the more heuristic DL methods and using data from bulk defleshing alone. The superiority of the DCNN method over traditional multivariate approaches (i.e., logistic regression) is shown by the latter having detected no difference between marks made on fleshed and defleshed bones^26^ and the former discriminating almost 95% of marks in both cases. It should be remarked that the multivariate LR method used categorical variables, whereas the DCNN method used pixel feature map numerical information. Both methods differ widely and so do their respective potentials.

The implementation of artificial intelligence methods to taphonomy has increased the resolution with which bone surface modifications are identified and classified^1^. The use of convolutional neural networks is a trend that is spreading in image-based analysis in several other fields, such as medicine^28–31^, botany^32^, economics^33^, geology and paleontology^34–36^. The combination of objectivity in data collecting and massive amounts of feature information opens the door to a new world of possibilities for understanding the intricacy of factors that determine taphonomic modifications of bones. We foresee a degree of accuracy in BSM classification never achieved before. Deep learning has landed in taphonomic research and it is here to stay.

## Supplementary information

Supplementary graphical information

## Data Availability

Code and images for the low-, intermediate- and high-resolution experiments are available in Harvard’s Dataverse public repository at https://dataverse.harvard.edu/dataset.xhtml?persistentId=doi%3A10.7910%2FDVN%2FYHKWMR.
