# Supplementary material for: Deep learning and taphonomy: high accuracy in the classification of cut marks made on fleshed and defleshed bones using convolutional neural networks
Source: Sci Rep. 2019 Dec 12;9:18933. doi: 10.1038/s41598-019-55439-6 (PMC6908723; doi:10.1038/s41598-019-55439-6)
Supplement: Supplementary file 1 — Supplementary graphical information [file 41598_2019_55439_MOESM1_ESM.pdf]

Deep learning and taphonomy: high accuracy in the classification of cut marks made on fleshed and defleshed bones using convolutional neural networks.

Gabriel Cifuentes-Alcobendas[1], Manuel Domínguez-Rodrigo*[1]

[1]IDEA (Institute of Evolution in Africa), University of Alcalá de Henares, Covarrubias 36, 28010 Madrid.

*Corresponding author

SUPPLEMENTARY INFORMATION

ADDITIONAL INFORMATION ABOUT THE METHOD AND SAMPLE USED

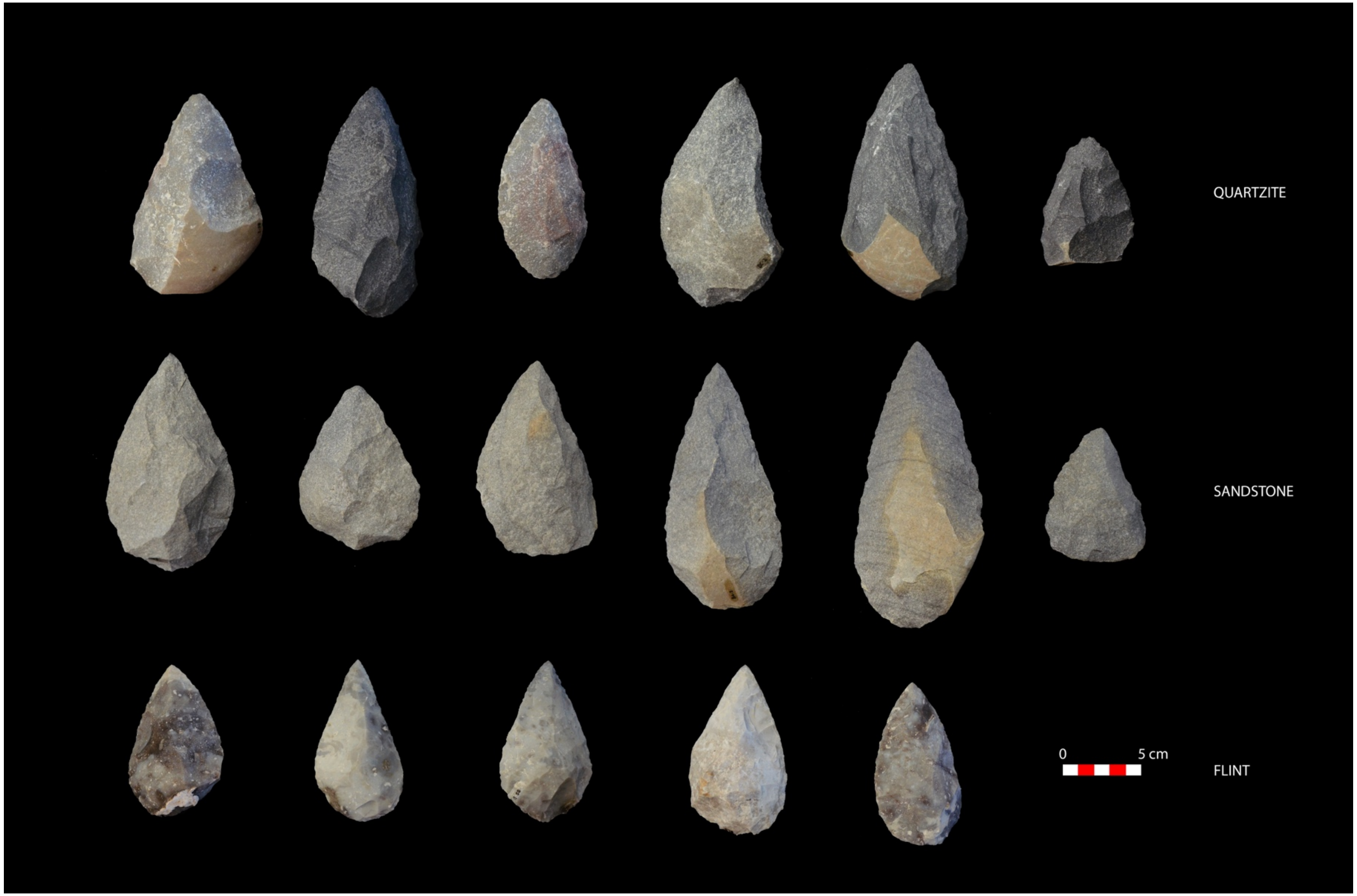

FIG S1. Types of handaxes used for both experiments (butchery and deflshed bones)

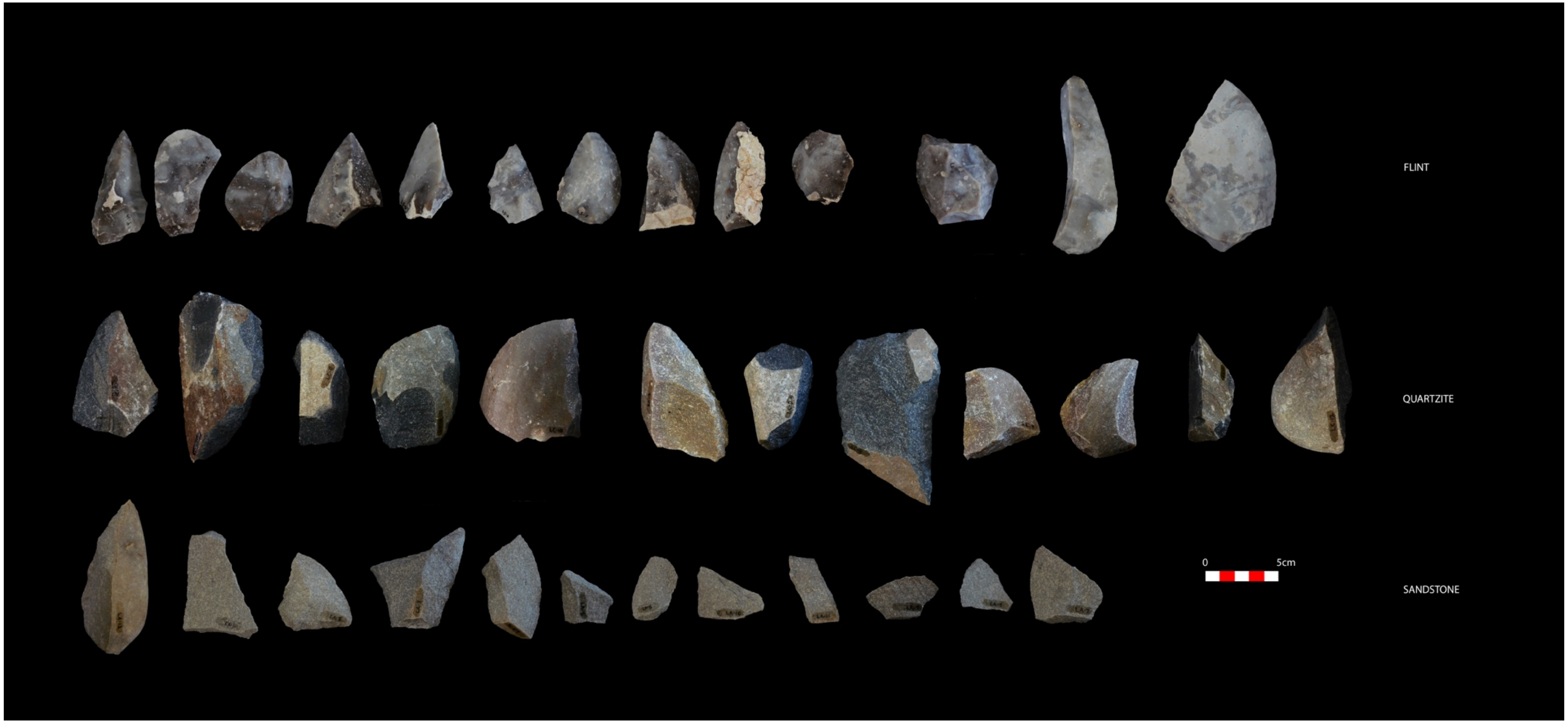

FIG S2. Types of flakes used for both experiments (butchery and deflshed bones)

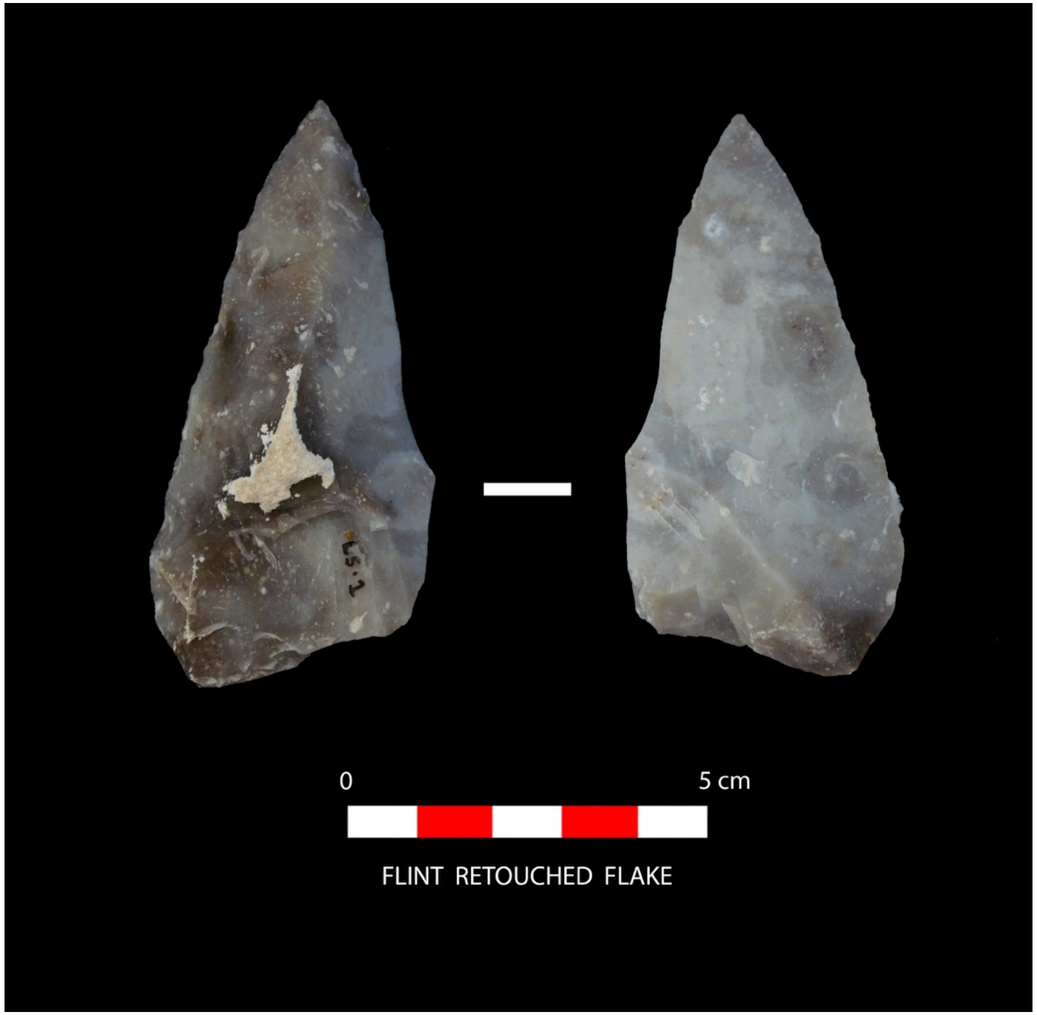

Fig. S3. Simple flake selected for the high-resolution experiment.

Table S1. Types of tools and raw materials used for each of the three experiments.

LOW RESOLUTION		
Used tools		
Handaxes	Chert	5
	Quartzite	6
	Arenisca	6
Retouched flakes	Chert	13
	Quartzite	12
	Sansdstone	12
Cut marks on defleshed bones	203	
Cut marks on bones with meat	1087	
Total number of cut marks	1290	

MODERATE RESOLUTION		
Used tools		
Retouched flakes	Chert	2
	Quartzite	3
Cut marks on defleshed bones	101	
Cut marks on bones with meat	132	
Total number of cut marks	233	

HIGH RESOLUTION		
Used tools		
Retouched flakes	Chert	1
Cut marks on defleshed bones	20	
Cut marks on bones with meat	28	
Total number of cut marks	48	
